# REMAINING SOCIALLY CONNECTED AT 100 AND BEYOND REDUCES IMPACT OF LONELINESS ON NUTRITIONAL STATUS

**DOI:** 10.1093/geroni/igz038.152

**Published:** 2019-11-08

**Authors:** Seung Eun Jung, Alex bishop, Seoyoun Kim, Janice Hermann

**Affiliations:** 1 University of Alabama, Tuscaloosa, Alabama, United States; 2 Oklahoma State University, Stillwater, Oklahoma, United States; 3 Texas State University, San Marcos, Texas, United States

## Abstract

Understanding factors influencing centenarians’ nutritional status can offer insight into effective nutrition interventions to improve quality of life among this population. This cross-sectional study was conducted to evaluate the moderating role of social support in the relationship between loneliness and nutritional status among Oklahoma centenarians (n=140). Nutritional status was assessed with the Mini Nutrition Assessment (MNA). Perceived social support was assessed with the 24-item Social Provisions Scale. Loneliness was examined with the 10-item UCLA loneliness scale. Ordinal logistic regression revealed that those who lacked social support were more likely to be at risk for malnutrition (OR=2.28, p<.05). Further, the interactive model revealed that centenarians who reported lack of support and loneliness were almost 2.8 times as likely to be at risk for malnutrition compared to their socially embedded counterparts (p<.01). Findings suggest that nutrition interventions offering centenarians opportunities to feel socially connected could improve their nutritional well-being.

